# ANCA-Negative Pauci-Immune Necrotizing Glomerulonephritis in the Setting of Multisystem Inflammatory Syndrome in Children

**DOI:** 10.1016/j.xkme.2026.101254

**Published:** 2026-01-10

**Authors:** Christine A. VanBeek, Naseem Alammar, Qassim Abid

**Affiliations:** 1 Ameripath Laboratories, Oklahoma City, OK; 2 Department of Pediatrics, Children’s Hospital at University of Oklahoma Health Sciences Center, Oklahoma City, OK; 3 Department of Nephrology, Children’s Hospital at University of Oklahoma Health Sciences Center, Oklahoma City, OK

**Keywords:** ANCA-negative pauci-immune necrotizing glomerulonephritis, crescentic glomerulonephritis, gross hematuria, multisystem inflammatory syndrome in children (MIS-C), pediatric COVID-19

## Abstract

Multisystem inflammatory syndrome in children (MIS-C) is a rare hyperinflammatory disorder that occurs in previously healthy pediatric patients after severe acute respiratory syndrome coronavirus 2 (SARS-CoV-2) exposure or mild infection. MIS-C typically has mild kidney symptoms that resolve quickly. The kidney biopsy experience in pediatric coronavirus disease 2019 (COVID-19) and MIS-C is limited in the literature. Here, we describe a 17-year-old SARS-CoV-2 positive boy with features of MIS-C who presented with abdominal pain and gross hematuria. He developed acute kidney injury requiring dialysis. Kidney biopsy and serologic work-up documented antineutrophil cytoplasmic antibody (ANCA)-negative pauci-immune necrotizing glomerulonephritis (AN-PING). Treatment with steroids and intravenous immunoglobulin resulted in simultaneous resolution of the systemic inflammatory markers and kidney symptoms. Aggressive immune suppression and cytotoxic agents were avoided. The glomerulonephritis remained in remission at 3-year follow-up. Although most cases of AN-PING are thought to represent kidney involvement by a primary vasculitis, a subset can occur in association with other conditions. We propose that AN-PING could be a rare kidney manifestation of MIS-C.

Pediatric severe acute respiratory syndrome coronavirus 2 (SARS-CoV-2) infections are typically mild or asymptomatic, with low hospitalization and mortality rates.^1^^,^^2^ The hyperinflammatory disorder, multisystem inflammatory syndrome in children (MIS-C), rarely develops in children and adolescents and is responsible for a subset of pediatric coronavirus disease 2019 (COVID-19) hospitalizations.^1^^,^^2^ Acute kidney injury (AKI) and urinalysis (UA) abnormalities may occur in hospitalized patients with pediatric COVID-19 or MIS-C, but kidney pathology data are limited.^3^^,^^4^ As a result, the histologic pattern and best management of kidney lesions in these patients is not well-established.

This case describes a 17-year-old boy with gross hematuria and severe AKI requiring dialysis because of ANCA-negative pauci-immune necrotizing glomerulonephritis (AN-PING) in the setting of SARS-CoV-2 infection. He had features of MIS-C, without COVID-19-related respiratory findings. Kidney symptoms improved quickly after steroid treatment, concurrently with MIS-C resolution.

## Case Report

The patient was a 17-year-old previously healthy boy who presented with a 1-week history of gross hematuria, fatigue, and constipation. Abdominal pain and emesis were noted 3 days prior but had resolved. Review of systems was otherwise negative, including no fevers, cough, sore throat, shortness of breath, nasal congestion, dysuria, changes in urinary output, or rash. He did not have any sick contacts and was not vaccinated for COVID-19. A family history of kidney disease requiring dialysis in his paternal grandfather was noted.

In the emergency department, the patient was hypertensive (blood pressure of 157/96 mm Hg) with pulse rate of 65, respiratory rate of 24, and temperature of 37.6 °C. Pulse oximetry was 100% on room air. The remainder of the physical examination was unremarkable.

Initial laboratory work-up (Table 1) showed a markedly elevated D-dimer levels as well as a serum creatinine (sCr) level of 7.85 mg/dL, hematuria, leukocyturia, and nonnephrotic proteinuria. Nasopharyngeal SARS-CoV-2 RNA polymerase chain reaction was positive. Urgent hemodialysis was initiated, and the patient received intravenous (IV) ceftriaxone.

**Table 1:** Laboratory Studies at Initial Presentation.

Test	Result	Reference Range
Hematology		
White blood cell count	10.96	(4-11 K/mm^3^)
Hemoglobin	13.1	(13.0-18.0 g/dL)
Hematocrit	39.3	(39-52.0%)
Platelet count	408	(140-440 K/mm^3^)
Coagulation		
D-Dimer	8,429 ↑^a^	<243 ng/mL DDU
Fibrinogen	389	(150-450 ng/mL DDU)
Prothrombin time	15.3 ↑^a^	(10-13 s)
INR	1.4 ↑^a^	(0.9-1.2)
Activated partial thromboplastin time	29.5	(26-37 s)
Chemistry		
BUN	160 ↑^a^	(7-17 mg/dL)
Creatinine	7.85 ↑^a^	(0.8-1.1 mg/dL)
Albumin	3.9	(3.8-5.1 g/dL)
Bilirubin	0.3	(0.3-1.2 mg/dL)
SGOT/AST	22	(8-41 Units/L)
ALT	16	(12-48 Units/L)
Total alkaline phosphatase	80	(79-291 Units/L)
Creatine kinase	73	(58-391 Units/L)
Lipase	57	(9-65 Units/L)
Cardiology		
Troponin	0.01	(0-100 pg/mL)
B-Natriuretic peptide	39	(0-100 pg/mL)
Infectious work-up^a^		
Anti-streptolysin O Ab	484 ↑^a^	(<32-250 U/mL)
Anti-DNase B (12/15/2021)	1,010 ↑^a^	0-170 U/mL
NP swab SARS-CoV-2 RNA PCR	Positive^a^	Negative
COVID 19 IgG Ab	Negative	Negative
Urine culture	*E. coli* positive^a^	No growth
Blood cultures	No growth	No growth
Hepatitis A antibody (IgM)	Nonreactive	Nonreactive
Hepatitis B surface antigen	Nonreactive	Nonreactive
Hepatitis B core antibody (IgM)	Nonreactive	Nonreactive
Hepatitis C antibody	Nonreactive	Nonreactive
HIV	Nonreactive	Nonreactive
Immunology		
C3	98	(83-157 mg/dL)
C4	17.3	(13-35 mg/dL)
CH50	>60	>41 U/mL
Rheumatoid factor	<10	(1-14 U/mL)
ANA	Positive (1:80 speckled)^a^	Negative
c-ANCA	<1:20	<1:20
p-ANCA	<1:20	<1:20
Atypical ANCA	<1:20	<1:20
Westergren ESR	37 ↑^a^	(0-15 mm/hr)
Interleukin 6	15.6 ↑^a^	(0-13 pg/mL)
Urine studies		
Urinalysis		
Protein	2+^a^	Negative
Blood	3+^a^	Negative
Red blood cells	>100^a^	0-2/high-power field
WBC	>100^a^	0-5/high-power field
Leukocyte esterase	2+^a^	Negative
Nitrite	Negative	Negative
Urine protein/creatinine ratio	1.32 ↑^a^	(<0.2 mg/mg)
Urine drug screen	Negative	Negative

Abbreviations: ALT, alanine aminotransferase; ANA, antinuclear antibody; ANCA, antineutrophil cytoplasmic antibody; AST, aspartate aminotransferase; B-NP, B-type natriuretic peptide; BUN, blood urea nitrogen; CH50, total hemolytic complement; COVID-19 IgG Ab, coronavirus disease 2019 immunoglobulin G antibody; C3, complement component 3; C4, complement component 4; D-dimer, fibrin degradation product; ESR, erythrocyte sedimentation rate; HIV, human immunodeficiency virus; INR, international normalized ratio; NP swab SARS-CoV-2 RNA PCR, nasopharyngeal swab severe acute respiratory syndrome coronavirus 2 ribonucleic acid polymerase chain reaction; RBC, red blood cell; SGOT, serum glutamic-oxaloacetic transaminase; WBC, white blood cell.

a Indicates abnormal results.

On kidney ultrasound, the kidneys measured 12.0 cm and 13.7 cm, and renal cortex had normal thickness with increased echogenicity. Abdominal ultrasound was negative for an acute process and lung bases did not have opacities. Transthoracic echocardiogram showed diffuse dilation of the left main coronary artery, without aneurysm or valvular vegetation. Serologic work-up showed a positive antinuclear antibody, anti-streptolysin O, and anti-DNase B. Antineutrophil cytoplasmic antibody (ANCA) screen, human immunodeficiency virus, and hepatitis panel were negative. Complements were normal. He continued to have markedly elevated inflammatory markers with a peak C-reactive protein levels of 95 mg/L, peak ferritin level of 872.4 ng/mL, peak calcitonin level of 12.07 ng/mL, neutrophilic leukocytosis (peak white blood cell count of 19.81 K/mm^3^), and peak D-dimer level of 18,152 ng/mL DDU. He developed fevers (up to 38.8 °C) and intermittent abdominal pain. Blood cultures were negative. There were no respiratory symptoms, and pulse oximetry remained within normal limits.

**Figure 1:**
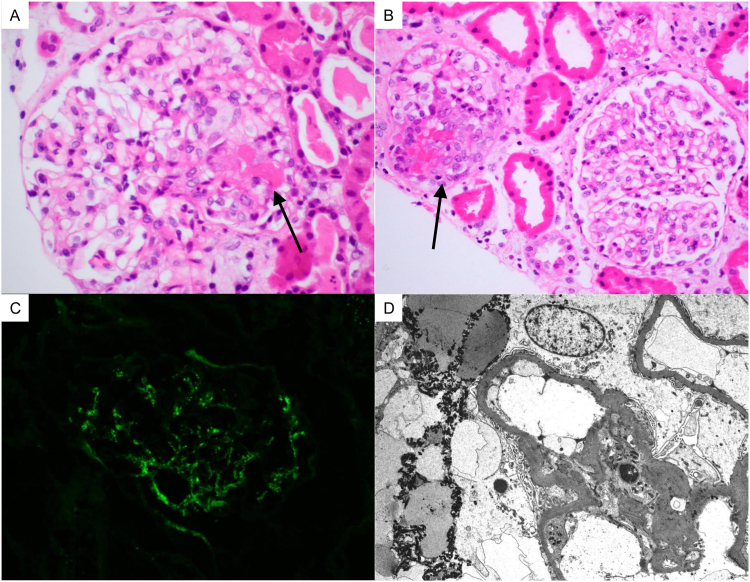
Biopsy findings. (A) Almost 50% of glomeruli in the biopsy had segmental necrotizing lesions (hematoxylin and eosin, original magnification 600×, arrow). (B) In a few glomeruli, there was early cellular crescent formation which is visible in the glomerulus on the left (arrow, hematoxylin and eosin, original magnification 400×). In addition, the uninvolved glomerulus on the right lacks hypercellularity. (C) There was mild to moderate mesangial C3 staining, with minimal to no staining for immunoglobulins, consistent with pauci-immune pattern (C3 immunofluorescence, original magnification ×400). (D) Fibrin tactoids are visible in the urinary space, but immune complex type deposits are sparse (electron microscopy, original magnification ×2,900).

A kidney biopsy was performed on hospital day 4. The light microscopy (LM) showed acute necrotizing glomerulonephritis (GN) involving 45% of glomeruli with early crescent formation in few glomeruli (Fig 1A and B). There was no intrinsic glomerular hypercellularity. One glomerulus (5% of total) was globally sclerotic. There was minimal tubular atrophy. Nonatrophic tubules demonstrated features of acute injury and had abundant red blood cell casts. The vessels were normal, without arteritis or microangiopathy. By immunofluorescence (IF), there was predominantly mesangial staining for C3 (2+) (Fig 1C), C1q (1+), IgA (trace to 1+), Kappa (trace), and Lambda (trace to 1+) in a granular pattern (grading scale of 0 to 4+). IgG and IgM were negative. There were sparse mesangial deposits by electron microscopy, without peripheral capillary wall deposits (Fig 1D). Few tubuloreticular inclusions were identified.

Because the patient met Centers for Disease Control and Prevention (CDC) criteria for MIS-C, he received 1 dose of intravenous immunoglobulin (IVIG) and 3 doses of IV methylprednisolone. His kidney function quickly recovered, and he was discharged a week after the biopsy with a sCr level of 1.4 mg/dL and D-dimer level of 3545/mL DDU. One week after discharge, his sCr was 1.17 mg/dL, although microhematuria was still detectable. He was asymptomatic and had negative UA at the 8-month and 3-year follow-ups. The most recent sCr level was 0.9 mg/dL.

## Discussion

MIS-C is a severe pediatric hyperinflammatory syndrome that typically occurs 2-6 weeks following mild SARS-CoV-2 infection or exposure.^5^^,^^6^ According to the updated case definition for MIS-C provided by the Council of State and Territorial Epidemiologists and CDC, clinical criteria include the following: (1) person aged less than 21 years, (2) subjective or documented fever, (3) illness with clinical severity requiring hospitalization or resulting in death, (4) evidence of systemic inflammation indicated by C-reactive protein ≥3.0 mg/dL, and (5) new onset manifestations in at least 2 categories (cardiac involvement, mucocutaneous involvement, shock, gastrointestinal involvement, or hematologic involvement).^5^ There also needs to be laboratory evidence of SARS-CoV-2 infection based on detection of SARS-CoV-2 RNA, SARS-CoV-2-specific antigen, or SARS-CoV-2-specific antibodies. Because MIS-C is a postinfectious phenomenon, detection of SARS-CoV-2 antibodies is more common than molecular detection of the virus.^5^

Given that MIS-C features are nonspecific and SARS-CoV-2 seroprevalence is increasing, it may be difficult to distinguish from alternative infectious or inflammatory disorders. Kawasaki Disease (KD) and severe acute COVID-19 are frequently considered in the differential diagnosis. The patient in our case met criteria for MIS-C because of gastrointestinal manifestations and cardiac involvement characterized by coronary artery dilatation. He did not have other signs of typical KD such as mucocutaneous lesions, lymphadenopathy, or conjunctival injection. His age is more similar to patients with MIS-C who tend to be older (median age of 9 years) than those with KD (typically under 5 years old).^7^^,^^8^ Pediatric patients with severe acute COVID-19 have more prominent pulmonary pathology and often have pre-existing comorbid conditions.^2^ In contrast, MIS-C is usually in previously healthy individuals and has pronounced gastrointestinal manifestations, similar to the current patient.^2^

The gross hematuria and AKI requiring dialysis described here would be unusual for MIS-C. Microscopic UA abnormalities have been noted in a subset of patients with MIS-C.^3^^,^^4^ There is 1 notable case of progressive severe hematuria with normal kidney function in the setting of MIS-C, with disappearance of kidney symptoms after IVIG treatment.^9^ AKI is relatively common in MIS-C but is typically mild.^3^^,^^4^ Hypotension, poor cardiac function, dehydration, or nephrotoxic agents have been proposed as potential contributing factors to MIS-C-associated AKI.^3^^,^^4^ Because AKI in MIS-C usually resolves quickly, kidney biopsies are generally not performed, and little is known about the corresponding histologic features.

Kidney biopsy LM documented acute necrotizing GN in this case. The modest IgA/C3 staining by IF was unlikely to be the etiology of the GN given the absence intrinsic glomerular hypercellularity and ultrastructural finding of sparse deposits limited to the mesangium. Based on minimal immunoglobulin staining, the IF pattern met previously established criteria for pauci-immune, and the lesion was classified as AN-PING.^10^ Although the vast majority of AN-PING cases are primary, a subset develops in other settings such as bacterial infection, autoimmune disease, malignancy, and drugs.^11^ The patient’s work-up for disorders that may contribute to secondary AN-PING was largely negative. Asymptomatic bacteriuria was noted, but this is not a well-documented cause of AN-PING or systemic hyperinflammation. The strong temporal association of kidney disease and MIS-C symptoms suggests that MIS-C may be another condition that can be associated with AN-PING. The pathogenesis of MIS-C and AN-PING are poorly defined, so the link between the 2 entities is not clear. However, investigators have highlighted the importance of neutrophil and monocyte activation in the development of both disorders.^11^^,^^12^ AN-PING and systemic hyperinflammation because of primary pauci-immune small vessel vasculitis (ANCA-associated vasculitis spectrum) with incidental SARS-CoV-2 positivity could be considered as an alternative explanation for the constellation of findings. Primary AN-PING has considerable risk of kidney disease progression and vasculitis relapse despite immunosuppression.^11^ In contrast, MIS-C commonly resolves after the initial hospitalization, as was seen in this patient.^7^

There are only isolated biopsy reports of de novo acute GN in MIS-C or pediatric patients with COVID-19. Rare examples of IgA vasculitis, acute thrombotic microangiopathy, pANCA-associated vasculitis, collapsing glomerulopathy, diffuse proliferative immune complex-mediated GN, and crescentic immune complex-mediated GN have been described.^13^, ^14^, ^15^, ^16^, ^17^ Because data are limited and heterogeneous, it can be difficult to determine whether the glomerular disease is truly related to the COVID-19. Kidney biopsies of patients with COVID-19 are negative with SARS-CoV-2 in situ hybridization, suggesting direct viral infection does not play a role in GN development.^18^ To our knowledge, AN-PING has not been previously reported in pediatric acute COVID-19 or MIS-C. Basiratnia et al^19^ documented 1 case of new onset ANCA-negative necrotizing GN in a patient who had similar clinical features including AKI briefly requiring dialysis, gross hematuria, gastrointestinal manifestations, and rapid improvement of kidney symptoms after steroids. However, an influx of neutrophils was noted by LM, and it is not certain if this was pauci-immune because IF was unavailable.

There were only 9,782 patients with MIS-C reported in US jurisdictions by the CDC as of August 4, 2025.^8^ Cases are decreasing with new phases of the pandemic and vaccine implementation but have not disappeared.^7^ Vigilance is needed in considering this potentially lethal condition when faced with a hyperinflammatory syndrome of unknown etiology. This case suggests that AN-PING can potentially coincide with MIS-C phenotype. Furthermore, the lesion may have an excellent prognosis after IVIG and methylprednisolone treatment in this particular setting.
